# Neuropsychiatric Systemic Lupus Erythematosus (NPSLE): A Case Report and an Overview of the Diagnosis, Treatment Modalities, and Prognosis

**DOI:** 10.7759/cureus.65593

**Published:** 2024-07-28

**Authors:** Ameena Syed, Sajid Shaik, Roshan Afshan, Andrew Karam, Wasif Hafeez, Sarmad Almansour

**Affiliations:** 1 Department of Internal Medicine, Orlando Health Physician Associates, Orlando, USA; 2 Department of Internal Medicine, Saint Vincent Hospital, Worcester, USA; 3 Department of Internal Medicine, Detroit Medical Center, Wayne State University, Sinai Grace Hospital, Detroit, USA; 4 Department of Emergency Medicine, Ascension Providence Hospital, Detroit, USA; 5 Department of Rheumatology, Medcare Hospital, Dubai, ARE

**Keywords:** anti-nucleic acid antibody (ana), complement 4, complement 3, anti-double stranded (anti-ds) dna, cerebral spinal fluid (csf), systemic lupus erythematosus (sle), neuropsychiatric systemic lupus erythematosus (npsle), american college of rheumatology (acr), npsle

## Abstract

Systemic lupus erythematosus (SLE) is a chronic inflammatory, multisystem autoimmune disease with a broad spectrum of clinical presentations. Neuropsychiatric systemic lupus erythematosus (NPSLE) refers to neurological and psychiatric symptoms involving the central and peripheral nervous systems.

A 23-year-old African American female with a history of undifferentiated connective tissue disease on hydroxychloroquine and poor medication adherence presented to the emergency department with an altered mental status and generalized headache. In addition, she had a fever, associated tachycardia (104 BPM), and hypotension (90/63 mmHg). She was given fluids and started on broad-spectrum antibiotics and antivirals, suspecting bacterial or viral meningitis. However, a broad infectious workup, including cerebral spinal fluid (CSF) culture, was unrevealing.

Given the lack of improvement of antibiotics, an immunological workup for SLE was initiated, which showed low CH50, C3, and C4; anti-nucleic acid antibody (ANA) was 1:1280, anti-double-stranded (anti-DS) DNA antibody not detected, and fluorescent ANA was positive. For severe NPSLE, rituximab is the most commonly utilized immunosuppressant; it was not utilized in this case due to the patient's insurance. The patient was placed on methylprednisolone and cyclophosphamide (CYC) infusion per ACR guidelines. Due to the toxic effects of CYC on the gonads, we offered ovarian preservation; however, the patient opted to refuse. The patient's mental status started to improve after three days of pulse steroids. The patient was advised to follow up with rheumatology for CYC therapy and a gradual taper of her steroids. NPSLE is a diagnosis of exclusion primarily based on expert opinion due to the absence of a gold standard diagnostic procedure. Disease-specific therapy, symptomatic therapy, nonpharmacological approaches, and correction of aggravating variables are all used to treat individuals with NPSLE.

This paper aims to contribute to the existing literature on NPSLE, with the intention to educate and strive for early detection and treatment. We hereby present an interesting case of SLE in a 23-year-old female who would not have responded to one treatment. Instead, she needed multidisciplinary management, along with poor compliance.

## Introduction

Systemic lupus erythematosus (SLE) is a chronic inflammatory, multisystem autoimmune disease. It has a waxing and waning course and a broad spectrum of clinical presentations [1]. The prevalence of SLE varies among populations and is approximately 50 in 100,000 [2]. Nonspecific fatigue, fever, arthralgia, and weight changes are the most common symptoms in new cases or recurrent flares [3]. In severe cases, it can advance to severe life-threatening diseases with multiorgan involvement, such as neuropsychiatric systemic lupus erythematosus (NPSLE), lupus pericarditis, or lupus nephritis (LN) [4].

According to meta-analysis, the prevalence of NPSLE among patients with SLE reaches up to 56% [2]. It is more commonly seen in African Americans, Hispanics, and Asians than in White individuals [5-7]. However, white patients have a higher incidence of neuropsychiatric manifestations [8,9]. The risk of developing NPSLE depends on genetic, environmental, and hormonal factors. Multiple pathogenic pathways like antibody-mediated neurotoxicity, vasculopathy due to anti-phospholipid (aPL) antibodies, cytokine-induced neurotoxicity, neuroplasticity loss, and other mechanisms are believed to be linked to specific clinical manifestations [2].

Approximately 90% of patients with NPSLE present with pure CNS manifestations; the most seen manifestations are headache (28.3%), mood disorders (20.7%), cognitive dysfunction (19.7%), seizures (9.9%), and cerebrovascular disease (8.0%) [2]. Other complications of SLE, like LN, affect approximately 40% of patients [5]. The prevalence of pericarditis is 39%, and the incidence ranges between 11% and 54% [6,7].

## Case presentation

A 23-year-old African American female in her twenties presented to the emergency department with altered mental status after she was found unresponsive by her mother. History was obtained from the patient's mother, who endorsed that the patient first developed a facial rash and mouth ulcers, followed by decreased appetite, and bilateral leg pain. This was followed by worsening generalized headache and a fever. She has a history of asthma, essential hypertension*, *and pericarditis status post-pericardial window placement. She was on 200 mg of hydroxychloroquinedaily but the patient discontinued the therapy within six months after she started to have blurry vision. On presentation, she was hypotensive with altered mental status. She later developed generalized tonic-clonic seizures, which were aborted with antiseizure medication. The patient had no history of seizures in the past. Initial computed tomography (CT) of the head without contrast was concerning for brain abscess.

However, the CT head was ill-defined, and the contrast showed multiple foci of ill-defined hypoattenuation without any ring enhancement. We followed up with an MRI, which revealed multiple small white-matter lesions. 

Blood culture, chest x-ray, and urine analysis were negative for infection. COVID-19, respiratory syncytial virus, influenza A, and influenza B were negative in the nasopharyngeal swab. White blood count remained within normal limits. She was treated symptomatically with fluids and started on acyclovir, ceftriaxone, vancomycin, and dexamethasone. The cerebrospinal fluid (CSF) analysis revealed a non-infectious process, as depicted in Table 1.

**Table 1:** CSF analysis CSF, cerebrospinal fluid

Cerebrospinal analysis	Lab value	Reference values
Glucose	35	40-70 (mg/dL)
Protein	177	15-45 (mg/dL)
Nucleated cells	26	0-5 (cm)
Neutrophils	43%	
Lymphocytes	37%	
Monocytes	20%	
Molecular biology	HSV-1 (negative); HSV-2 (negative)	
CSF cultures	Negative	

Immunological workup for SLE showed CH50 was 36 mg/dL (low, normal: 42-91 mg/dL), C3 was 31 mg/dL (low, normal: 87-200 mg/dL), C4 was less than 8 mg/dL (low, normal: 19-52 mg/dL), ANA was 1:1280, anti-double-stranded (anti-DS) DNA antibody was not detected, fluorescent anti-nucleic acid antibody (ANA) was positive. Antibiotics and antivirals were discontinued after a lack of response to therapy. The patient was started on methylprednisone (1 mg/kg/day) and cyclophosphamide (CYC) infusion (per National Institute of Health Protocol) for concerns of active lupus cerebritis. The patient's mentation improved after three days of pulse steroids, with dramatic improvement after the first dose of CYC. The patient was discharged home with an oral steroid taper regimen and plans to continue CYC infusion as an outpatient. 

On subsequent follow-up by rheumatology, the laboratory results revealed that the patient had severe proteinuria. Urinalysis showed 2+ protein, urine creatinine was 66.3 mg/dL (normal: 29-226 mg/dL), urine protein was found to be 515 mg/dL (normal: <12 mg/dL), and GFR was 76 (chronic kidney disease stage 2). As we were concerned about possible LN, we advised renal biopsy, which the patient refused. 

## Discussion

NPSLE is a diagnosis of exclusion primarily based on expert opinion due to the absence of a gold standard diagnostic test [1]. NPSLE is a constellation of neurological and psychiatric symptoms involving the central and peripheral nervous systems [1]. Early presenting features in lupus-cerebritis can be potentially misleading and create a significant diagnostic dilemma [4]. Common neurologic manifestations include intractable headaches, seizures, demyelinating syndromes like multiple sclerosis, chorea, and aseptic meningitis. Approximately 10% to 15% of NPSLE cases have peripheral nervous system symptoms. The most common symptom is peripheral neuropathy, which encompasses mono- or polyneuropathy, cranial neuropathy, inflammatory demyelinating polyradiculoneuropathy, and plexopathy. Psychiatric manifestations include depression, anxiety, and psychosis. Neuropsychiatric syndromes observed in SLE are illustrated in Table 2 [10]. Alternative factors such as infection, concurrent disease processes, metabolic abnormalities, or pharmacological side effects must be ruled out first [8].

**Table 2:** Neuropsychiatric syndromes observed in SLE CNS, central nervous system; SLE, systemic lupus erythematosus () Author Credit: American College of Rheumatology Ad Hoc Committee

Neurological syndromes	CNS		Peripheral nervous system
	Focal	1. Aseptic meningitis	1. Guillain-Barré
		2. Cerebrovascular disease	2. Autonomic disorder
		3. Demyelinating syndrome	3. Mononeuropathy (single/multiplex)
		4. Headache	4. Myasthenia gravis
		5. Movement disorder	5. Cranial neuropathy
		6. Myelopathy	6. Plexopathy
		7. Seizure disorders	7. Polyneuropathy
Psychiatric syndromes	Diffuse	8. Acute confusional state	
		9. Anxiety disorder	
		10. Cognitive dysfunction	
		11. Mood disorder	
		12. Psychosis	

Circulating autoantibodies

Among all circulating autoantibodies, anti-aPL, anticardiolipin (aCL), lupus anticoagulant (LAC), and beta 2-glycoprotein antibodies provide the most diagnostic information in NPSLE, particularly in individuals with focal NPSLE events such as cerebrovascular illness and seizures. It is proposed that serum anti-ribosomal P-antibodies may be linked to lupus psychosis.

Because of the lack of specificity, measuring autoantibodies or cytokines in CSF is not suggested in clinical practice [11]. Despite significant progress, specific laboratory and neuroimaging biomarkers for NPSLE are uncommon, and their validation as valuable biomarkers in ordinary clinical practice is still a long way off [12].

CSF analysis

Patients with NPSLE may have a typical CSF composition, anti-DNA antibodies, oligoclonal banding, immune complexes, and interleukin-6, and B-cell activation indicators have been found in the CSF of NPSLE patients. Our study was limited in that we could not check for anti-DNA antibodies and oligoclonal banding as our initial suspicion was only meningitis. 

Neuroimaging

**Figure 1:**
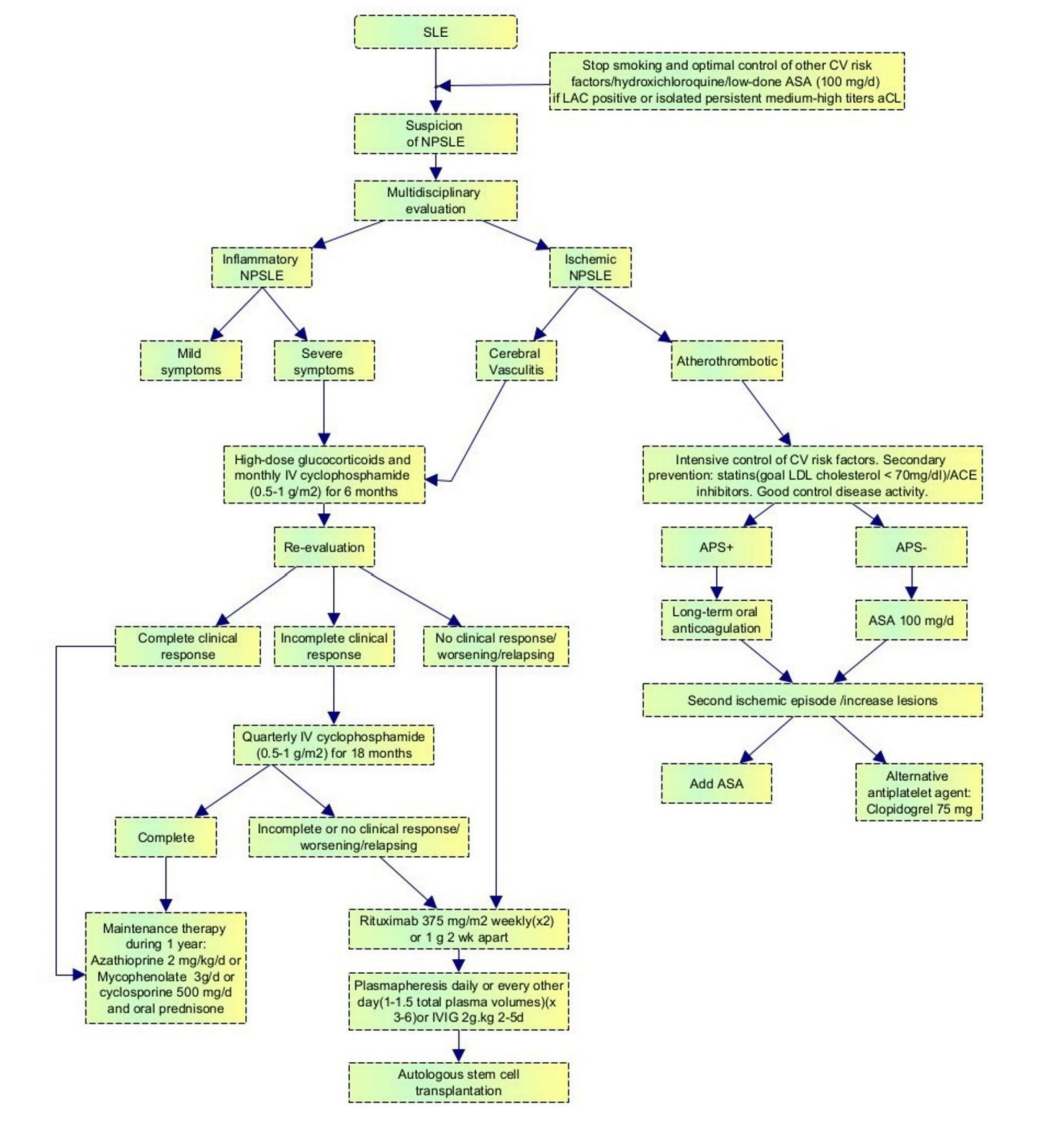
Diagnostic algorithm for patients presenting with signs and symptoms suggestive of NPSLE ASA, aspirin; APS, antiphospholipid syndrome; aCL, anticardiolipin; LAC, lupus anticoagulant; IVIG, intravenous Immunoglobulin; CV, cardio vascular; NPSLE, neuropsychiatric systemic lupus erythematosus

CT is primarily utilized in emergency settings to rule out specific abnormalities such as infarcts, bleeding, and malignancies. The neuroimaging technique of choice in NPSLE is magnetic resonance imaging (MRI), which identifies NPSLE-related lesions such as infarcts or myelopathy. The diagnostic algorithm for patients presenting with signs and symptoms suggestive of NPSLE is illustrated in Figure 1 [1].

Even though they are rare, neuropsychiatric manifestations could be the first presenting symptoms of NPSLE. Therefore, the threshold for suspecting NPSLE should be very low in patients with non-specific neuropsychiatric symptoms. We recommend a multidisciplinary team, which includes rheumatology, internal medicine, neurology, and nephrology* *to diagnose and manage patients with long-standing SLE and multisystem complications. These patients are always treated with a general therapeutic approach like that used in non-SLE patients with the same neuropsychiatric presentation. 

Disease-specific therapy, symptomatic therapy, nonpharmacological techniques, and correction of aggravating variables are all used to treat individuals with NPSLE. Treatment should be catered to the patient's specific needs, including suspected mechanisms (such as evidence of thrombosis or the presence of specific antibodies), the severity of symptoms, expected morbidity, time since onset of symptoms, reversibility, response to previous therapies, and impact on quality of life [1]. 

Disease-specific therapy

Immunosuppressive medications, such as systemic glucocorticosteroids (GC), can produce a positive response in 60% to 75% of patients.Pulse therapy with very high doses of GC (methylprednisone 1 mg/kg/day) followed by oral therapy is widely used for severe signs or symptoms [1].

In many moderate to severe NPSLE cases, additional immune suppressants are required to control the condition and allow GC discontinuation. Several studies have shown that azathioprine and mycophenolate mofetil can be used as second-line and maintenance therapy to avoid extended exposure to high-dose steroids or replace CYC therapy [1,2]. Other methods like anti-B cell therapy (rituximab anti-CD20 monoclonal antibodies), belimumab (a monoclonal antibody directed against B cell-activating factor), plasma exchange, and intravenous immunoglobulin can be considered in cases with poor response or failure of standard treatment [13]. Plasmapheresis has been demonstrated to be beneficial as an additional therapy for chorea or myelitis in NPSLE patients [14].

Anti-aggregation/anticoagulation therapy

It is usually recommended when symptoms are localized and clinical and radiographic evidence of ischemia or thrombotic events are present. The current suggestion is to treat individuals with SLE who are seropositive for aPL antibodies with antiaggregant as the main prevention strategy, with anticoagulants used as a secondary prevention strategy [15,16].

Symptomatic therapy

Symptomatic therapy is required in several NPSLE disorders. Antidepressants, antipsychotics, and anxiolytics are used for psychiatric diseases based on conventional indications. When high-risk symptoms such as a second seizure (>24 hours after the first occurrence), substantial brain injury, brain structural abnormalities (MRI), focal neurological indications, partial seizure as the initial seizure, or epileptiform electroencephalogram discharges are present, anti-epileptic therapy is initiated [1]. Dopamine agonists are used as a symptomatic treatment for movement disorders. Nonsteroidal anti-inflammatory medications (NSAIDs) can alleviate symptomatic pain and migraine headaches.

Nonpharmacological intervention

Psychosocial factors such as exhaustion, sleep deprivation, pain, depression, and anxiety have been linked to cognitive impairment. In SLE, there is no precise evidence-based symptomatic therapy for cognitive dysfunction. However, some people with depression and cognitive dysfunction may benefit from antidepressants. For example, an eight-week psychoeducational group intervention improved SLE patients' coping skills and, as a result, their quality of life significantly and sustainably improved [1].

Control of aggravating factors

Controlling precipitating variables such as hypertension, infection, metabolic imbalances, valvular disease, and adverse medication effects is critical. Neurological involvement in SLE and its complications worsen the prognosis of the disease and treatment outcome [17].

## Conclusions

In patients with a history of connective tissue disorders who present with neurological symptoms, it is imperative to maintain a high index of suspicion for lupus cerebritis. Prompt consideration of this diagnosis is crucial to avoid delays, preserve critical time, and mitigate associated mortality and morbidity.
